# Fermenting Acerola (*Malpighia emarginata* D.C.) and Guava (*Psidium guayaba* L.) Fruit Processing Co-Products with Probiotic Lactobacilli to Produce Novel Potentially Synbiotic Circular Ingredients

**DOI:** 10.3390/foods13091375

**Published:** 2024-04-29

**Authors:** Caroliny M. Araújo, Thatyane Mariano R. de Albuquerque, Karoliny B. Sampaio, Jordana N. de Oliveira, Jaielison Yandro P. da Silva, Marcos dos S. Lima, Yuri M. do Nascimento, Evandro F. da Silva, Marcelo S. da Silva, Josean F. Tavares, Evandro L. de Souza, Maria Elieidy G. de Oliveira

**Affiliations:** 1 Laboratory of Food Microbiology, Department of Nutrition, Health Sciences Center, Federal University of Paraíba, João Pessoa 58051-900, Brazil; carolnutripb@gmail.com (C.M.A.); thatyane.albuquerque3@academico.ufpb.br (T.M.R.d.A.); karolbsampaio@gmail.com (K.B.S.); jordana.nunes@academico.ufpb.br (J.N.d.O.); jaielison.yandro@academico.ufpb.br (J.Y.P.d.S.); els@academico.ufpb.br (E.L.d.S.); 2 Department of Food Technology, Federal Institute of Sertão Pernambucano, Petrolina 56302-100, Brazil; marcos.santos@ifsertao-pe.edu.br; 3 Institute for Research in Drugs and Medicines—IPeFarM, Federal University of Paraíba, João Pessoa 58051-900, Brazil; yurimangueira@ltf.ufpb.br (Y.M.d.N.); evandro@ltf.ufpb.br (E.F.d.S.); marcelosobral@ltf.ufpb.br (M.S.d.S.); josean@ltf.ufpb.br (J.F.T.); 4 Laboratory of Food Bromatology, Department of Nutrition, Health Sciences Center, Federal University of Paraíba, João Pessoa 58051-900, Brazil

**Keywords:** fruit waste, probiotic, prebiotic, gut microbiota, functional ingredient, metabolism

## Abstract

This study evaluated the effects of acerola and guava fruit processing co-products fermented with probiotic *Lactobacillus acidophilus* LA-05 and *Lacticaseibacillus paracasei* L-10 on the abundance of different intestinal bacterial groups and microbial metabolic activity during 48 h of in vitro fecal fermentation. Digested fermented fruit co-products increased the relative abundance of beneficial bacterial groups while overall decreasing or maintaining the relative abundance of non-beneficial bacterial groups, suggesting selective stimulatory effects on beneficial bacterial intestinal populations. The fermented co-products stimulated microbial metabolic activity due to decreased pH, sugar consumption, short-chain fatty acid production, phenolic compound and metabolic profile alteration, and high antioxidant capacity during fecal fermentation. Acerola and guava co-products have high nutritional value and bioactive compounds whose fermentation with probiotics improves their potential functionalities. The results show that fermented fruit co-products could induce beneficial changes in the relative abundance of several bacterial groups as well as in the metabolic activity of the human intestinal microbiota. These results highlight their potential as novel and circular candidates for use as synbiotic ingredients.

## 1. Introduction

Fruits and fruit-derived food products have attracted greater consumer interest in recent decades due to their nutritional content and bioactive components associated with several health-promoting properties [1]. Brazil is one of the world’s largest tropical fruit producers. Acerola (*Malpighia emarginata* D.C.) and guava (*Psidium guajava* L.) are among the most popular tropical fruits cultivated in Brazil, being recognized for their high acceptance, nutritional value, variety of bioactive compounds, and reported beneficial effects on consumer health [2,3].

Most of the acerola and guava production is destined for processing to produce juices, frozen pulp, sweets, and jellies, with the generation of high quantities of agro-industrial co-products [4,5]. Reusing these co-products represents a relevant strategy for the circular economy linked to the agro-food sector, promoting potential circular ingredients that are characterized by prolonging the food life cycle, reducing waste, and making wise use of the available resources [6]. Acerola and guava co-products consist mainly of peels, seeds, and the remaining part of the pulp, representing a natural source of several bioactive compounds, including ascorbic acid, phenolic compounds, carotenoids, and dietary fiber, that could be exploited to produce synbiotic functional ingredients for the food and pharmaceutical industry due to their well-known antioxidant and/or potential prebiotic properties [7,8,9,10].

Prebiotics are recognized as compounds that resist gastric acidity, are not hydrolyzed by the upper part of the gastrointestinal tract, and reach the colon to be fermented by the beneficial intestinal microbiota, including probiotics, to provide beneficial effects on health, such as improving digestive system function and immunological response [11,12]. Furthermore, phenolic compounds found in fruit co-products are metabolized by the colonic microbiota and exert different physiological effects, including a local effect on modulating the intestinal microbiota [5,13,14].

Studies on the development of fruit co-products fermented by probiotics, as well as those highlighting their effects on the human intestinal microbiota for use as a synbiotic product, are still scarce. Few studies have evaluated in vitro the potential prebiotic properties of fruit co-products, including acerola and guava co-products, reporting stimulatory effects on the growth and metabolism of probiotic *Lactobacillus* and *Bifidobacterium*, in addition to increasing the production of short-chain fatty acids [15,16]. *Lactobacillus* spp. is typically added to commercial probiotic and fermented products due to its reported health benefits and safe human use [17]. There is a need to select appropriate probiotic strains for fermentation, and *Lactobacillus acidophilus* and *Lacticaseibacilus paracasei* (previously referred to as *Lactobacillus paracasei*) are among the *Lactobacillus* species most widely used as probiotics and linked to improvements in intestinal microbiota [18], in addition to having satisfactory fermentative abilities [19,20].

The *Lactobacillus* genus is commonly used as starters and co-starters in producing fermented products with good survival in low pH products during fermentation and refrigerated storage [21]. The fermentation of acerola and guava co-products with probiotic lactobacilli has been shown to be an effective strategy to improve their physicochemical characteristics, bioactive compound profiles, and antioxidant properties [22]. Several factors have been linked to the emergence of fruit-based fermented products as carriers of probiotics, such as lactose intolerance, sensitivity to milk protein, and adoption of specific diet patterns (e.g., vegan, paleo, and flexitarian) [23]. The combination of potentially prebiotic fruit co-products and probiotic strains could represent an advantage in producing novel synbiotic circular ingredients, which could be value-added foods that are more effective than either probiotics or prebiotics alone in beneficially affecting the intestinal microbiota and inducing physiological outcomes [20,24].

This study hypothesized that the fermentation of acerola and guava co-products with probiotics could produce novel synbiotic ingredients with beneficial effects on human intestinal microbiota. To test this hypothesis, this study performed a submerged fermentation of acerola and guava co-products with probiotic *L. acidophilus* and *L. paracasei* strains and evaluated the effects of the obtained fermented co-products on the abundance of different bacterial populations and metabolic activity of human intestinal microbiota during an in vitro fecal fermentation with a pooled human fecal inoculum. 

## 2. Materials and Methods

### 2.1. Preparation of Fruit Processing Co-Products

The co-products (peel, seeds, and pomace) of acerola (*Malpighia emarginata* D.C.) and guava (*Psidium guayaba* L.) were obtained from a fruit pulp processing industry (João Pessoa, PB, Brazil). The samples were collected from four fruit pulp processing batches, totaling approximately 4 kg for each fruit. Each type of fruit co-product was separately packaged in plastic bags, frozen at −18 °C for 24 h, and freeze-dried (−55 °C, vacuum pressure of <138 μHg, freeze-drying rate of <1 mm/h, approximately 12 h) using a benchtop freeze dryer (model LI-101; LIOTOP^®^, São Carlos, Brazil). The freeze-dried material was grounded using a domestic blender (low speed for 10 min) and sieved through a fine mesh to obtain a powder with an average particle size of <1.0 mm. The powdered product was stored at −18 ± 2 °C in hermetically sealed polypropylene bags. The physicochemical characteristics of acerola and guava processing co-products were reported in previous studies [4,22], highlighting the high contents of soluble and insoluble fiber, strong antioxidant capacity, and presence of a wide variety of phenolic compounds, as summarized in Table S1 (Supplementary Materials).

Freeze-dried fruit co-products were individually suspended in distilled water (5% *w*/*v*), homogenized using an Ultra Turrax T25 (IKA, Staufen im Breisgau, Germany), and autoclaved (1.5 atm, 15 min, 121 °C).

### 2.2. Microorganisms and Fermentation Conditions

Two well-known probiotic strains, *Lactobacillus acidophilus* LA-05 and *Lacticaseibacillus paracasei* L-10 [25,26,27], were used in this study. Stock cultures were maintained in de Man, Rogosa, and Sharpe (MRS; HiMedia, Mumbai, India) broth containing glycerol (150 g/L) at −20 °C. For inoculum preparation, the strains were anaerobically cultured (Anaerogen System Anaerogen, Oxoid Ltd., Basingstoke, UK) in MRS broth at 37 ± 0.5 °C for 24 h. The resulting culture was inoculated (1%, *v*/*v*) in fresh MRS broth and incubated under the same conditions until reaching the stationary growth phase (20–24 h) [4]. These suspensions had viable cell counts of approximately 10 log CFU/mL [with optical density (OD) reading at 625 nm (OD_625_) corresponding to 1.5]. The probiotic suspensions were inoculated individually in 100 mL of acerola or guava by-product suspension with an inoculum concentration of 2% (*v*/*v*), followed by incubation at 37 ± 0.5 °C for 20 h with agitation (200 rpm). The samples with 20 h of fermentation were selected considering the results of preliminary experiments, which showed that the highest viable cell counts (i.e., approximately 9 log CFU/mL) of the tested probiotics in acerola and guava co-product suspensions were reached at this fermentation time (late exponential growth phase), followed by a decline in viable cell counts over time. 

### 2.3. Freeze-Drying of Fermented Fruit Co-Products 

Aliquots (10 mL) of fermented acerola or guava co-product suspensions were neutralized with NaOH to pH 7 to reduce stress on the bacterial cells during the drying process, transferred aseptically to glass vials, frozen at −18 °C for 24 h, and freeze-dried for 40 h (−55 ± 2 °C; vacuum pressure < 138 μHg, freeze-drying speed of 1 mm/h) using a bench-top freeze-dryer (model L-101, model L-101, LIOTOP®, São Carlos, Brazil) [28]. The fermented acerola and guava co-products had probiotic viable cell counts of >9 log CFU/mL when inoculated in MRS agar (HiMedia, Mumbai, India) at 37 ± 0.5 °C for 24 h. The freeze-dried fermented acerola and guava co-products were stored in desiccators with silica gel (4 ± 0.5 °C) [29]. Four distinct freeze-dried fermented suspensions were produced: (i) acerola co-product + *L. paracasei* L-10, named AL10; (ii) acerola co-product + *L. acidophilus* LA-05, named ALA5; (iii) guava co-product + *L. paracasei* L-10, named GL10; and (iv) guava co-product + *L. acidophilus* LA-05, named GLA5. The different freeze-dried fermented co-product suspensions were tested separately in the experiments.

### 2.4. Simulated Gastrointestinal Digestion of the Fermented Co-Product Suspensions

AL10, ALA5, GL10, and GLA5 were exposed to simulated gastrointestinal digestion following previously described procedures [30]. Initially, 10 g of each AL10, ALA5, GL10, and GLA5 were rehydrated (15 min) with sterile distilled water (50 mL, 25 ± 0.5 °C), and simulations were carried out continuously in glass flasks (250 mL) mimicking oral, gastric, and intestinal conditions using an incubator (37 ± 0.5 °C) and mechanical agitation to simulate the peristaltic movements; α-amylase (100 U/mL) diluted in 1 mM CaCl_2_ and pH adjusted to 6.9 with 1 M NaHCO_3_ (exposure time 2 min, 37 ± 0.5 °C, 200 rpm) to simulate the mouth conditions; and pH adjusted to 2.0 with 1 M HCl to simulate the stomach conditions. A pepsin solution (25 mg/mL) in 0.1 M HCl (proportion 0.05 mL/mL of the sample, exposure time 120 min, 37 ± 0.5 °C, 130 rpm) simulated the gastric juice. pH adjusted to 6.0 with 1 M NaHCO_3_ simulated the intestinal conditions, and 2 g/L of pancreatin and 12 g/L of bile salts diluted in 1 M NaHCO_3_ (proportion 0.25 mL/mL of the sample, exposure time 120 min, 37 ± 0.5 °C, 45 rpm) simulated the intestinal juice. 

The suspensions containing the final digestion phase were dialyzed against 0.01 mol/L NaCl (18 h, 5 ± 0.5 °C) with a regenerated cellulose dialysis tubing (1 kDa nominal molecular weight cut-off, Spectra/Por 6, Spectrum Europe BV, Breda, Netherlands). The dialyzed material was frozen (−18 °C), freeze-dried, and stored (5 ± 0.5 °C) in hermetically sealed polyethylene bags for a maximum period of one week [31]. The cellulose dialysis tubing, enzymes, bile salts, and reagents were purchased from Sigma-Aldrich (St. Louis, MO, USA).

### 2.5. Preparation of Human Fecal Inoculum and In Vitro Fecal Fermentation

An Ethical Committee on Research with Human Beings approved the procedures used in this study (Federal University of Paraíba, Joao Pessoa, PB, Brazil; protocol number 6.080.926), which followed the guidelines of the National Health Council (Resolution 466, 2012). Six healthy adult volunteers (three men and three women, ages 28 to 33) donated feces for the study and reported eating an omnivorous diet, not suffering from any gastrointestinal or colonic illness, not using concentrated probiotics or prebiotics, and not using antibiotics or other controlled medications for at least 6 months before feces collection [30,31,32,33].

Fecal samples were obtained using sterile tubes placed in an anaerobic container (Anaerogen System Anaerogen, Oxoid Ltd., Basingstoke, UK), pooled (1:1:1:1:1:1 *w*/*w*), homogenized with sterile phosphate-buffered saline (PBS; 0.1 M; pH 7.4; 1:10, *w*/*v*, 200 rpm, 2 min), and filtered using sterile triple-layer gauze to remove larger particles [32]. The fecal fermentation system was made using separated sterile tubes (50 mL working volume) containing 20% of digested and freeze-dried AL10, ALA5, GL10, and GLA5 (*w*/*v*); 40% of pooled fecal inoculum; and 40% of sterile (autoclavation, 121 °C, 1 atm, 15 min) fermentation medium (*v*/*v*) [composition: 4.5 g NaCl, 4.5 g KCl, 1.5 g NaHCO_3_, 0.69 g MgSO_4_, 0.8 g L-cysteine, 0.5 g KH_2_PO_4_, 0.5 g K_2_HPO_4_, 0.4 g bile salt, 0.08 g CaCl_2_, 0.005 g FeSO_4_, 1 mL Tween 80, 4 mL resazurin solution (0.25 g/L, as an anaerobic indicator), and 1 L of distilled water]. Each tube was placed inside an anaerobic jar equipped with an anaerobiosis generator system (Anaerogen System Anaerogen, Oxoid Ltd., Basingstoke, UK) and incubated for 48 h at 37 ± 0.5 °C to promote fermentation. A fecal fermentation system containing the well-known prebiotic fructooligosaccharides [FOS (not digested); 20%, *w*/*v*] and with no substrate added were included in the experiment as a positive and negative control, respectively [32,33,34]. Ingredients to prepare the fermentation media were purchased from Sigma-Aldrich, St. Louis, MO, USA.

#### 2.5.1. Enumeration of the Intestinal Bacterial Populations during In Vitro Fecal Fermentation

The capacity of AL10, ALA5, GL10, and GLA5 to affect the relative abundance of several human intestinal bacterial populations was assessed using fluorescent in situ hybridization combined with flow cytometry. The fluorescent dye Cy3 (Sigma-Aldrich, St. Louis, MO, USA) was used to identify six different probes: Lab 158 to enumerate *Lactobacillus* spp./*Enterococcus* spp., Bif 164 to enumerate *Bifidobacterium* spp., Rfla 729 to enumerate *Ruminococcus albus*/*R. flavefaciens*, Bac 303 to enumerate *Bacteroides* spp./*Prevotella* spp., Chis 150 to enumerate *Clostridium histolyticum*, and Erec 482 to enumerate *Eubacterium rectale*/*Clostridium coccoides*. SYBR Green staining (Molecular Probes, Invitrogen, Carlsbad, CA, USA) was used to enumerate the total bacterial population [15,16,32,33].

Aliquots (375 µL) from each medium were taken at time zero (immediately following homogenization of the components of the fermentation medium) and 24 and 48 h of fermentation, fixed overnight (4% paraformaldehyde), and hybridized with the fluorescent probes using previously described procedures [35,36]. The different bacterial populations were enumerated using a flow cytometer (BD Accuri C6, BD Biosciences, East Rutherford, NJ, USA). The BD Accuri C6 Software captured signals from individual cells (logarithmic signals) by passing through the laser zone, and fluorescence signals were recorded as cytograms on FL1 (SYBR Green) and FL2 (Lab 158, Bif 164, Rfla 729, Bac 303, Chis 150, and Erec 482). The results were expressed as the relative abundance (percentage) of cells hybridized with each probe concerning the total bacterial population [15,16,33,35].

#### 2.5.2. Determination of Microbial Metabolism during In Vitro Fecal Fermentation

Measurements of pH values and metabolic global profiling at time zero and 24 and 48 h of in vitro fecal fermentation were used to assess the intestinal microbiota metabolic activity in the media with AL10, ALA5, GL10, GLA5, FOS (positive control), and NC. The pH values (method 981.12) were determined with a digital potentiometer (Quimis, Diadema, SP, Brazil) [37]. Contents of sugars (glucose, fructose, maltose, and rhamnose), lactic and short-chain fatty acids (SCFA) (acetic, propionic, and butyric acids), and phenolic compounds were determined with high-performance liquid chromatography using a liquid chromatograph (model 1260 Infinity LC, Agilent Technologies, St. Clara, CA, USA) and previously described analytical conditions [38,39]. The OpenLab CDS, ChemStation Edition. Rev. C.01.10 (201) software (Agilent Technologies) was used to process the data. HPLC sample peaks were identified by comparing their retention times with sugar and organic acid standards (Sigma Aldrich). Average peak areas were considered to quantify sugars and organic acids [34,38].

The global metabolic profile of the fermentation media was analyzed using nuclear magnetic resonance (NMR). An aliquot (2 mL) of each fermentation medium was diluted in a solution (2 mL) with methanol and deuterated water (9:1, *v*/*v*) and filtered, and the resulting solution (550 μL) was placed in a tube (5 mm diameter) for NMR analysis. NMR experiments were performed with a Bruker Avance Neo 500 instrument (500 MHz for ^1^H NMR and 125 MHz for ^13^C NMR; Bruker, Billerica, MA, USA). The parameters to obtain the spectrum sequence were lc1pngpf2, temperature 26 °C, number of scans 64, Dummer scan 4, receiver gain 32, and acquisition time 3.27 s. The Bruker TopSpin 4.1.1 software was used to process the spectra [30,33].

#### 2.5.3. Determination of the Antioxidant Capacity during In Vitro Fecal Fermentation 

The antioxidant capacity in the media with AL10, ALA5, GL10, and GLA5 was measured with DPPH^•^ (2,2-diphenyl-1-picrylhydrazyl), FRAP (ferric reducing antioxidant power), and ABTS (2,2′-azino-bis (3-ethylbenzothiazoline-6-sulfonic acid) methods at time zero and 24 and 48 h of fecal fermentation. Initially, 2 g of media with AL10, ALA5, GL10, and GLA5 were homogenized separately with 10 mL of 80% methanol (Sigma-Aldrich, St. Louis, MO, USA), kept resting for 24 h under room temperature, and filtered (125 mm filter, Whatman®, GE Healthcare, Chicago, IL, USA). The determination of the antioxidant capacity using DPPH^•^ radical was performed as previously described [39]. Aliquots (50 µL) of the samples were reacted with DPPH solution in methanol (250 µL), shaken vigorously, and kept (30 min) in the dark. Measurements of DPPH scavenging activity were performed at a wavelength of 517 nm. Controls were prepared with water to replace a sample. For the “blank”, only the extracting solution (300 µL) was used. The antioxidant capacity was calculated using a 2 mM Trolox standard curve (10–2000 µM), and the inhibition percentages were determined using the following equation [40]:

DPPH radical scavenging capacity (%) = [(ABS control − ABS sample)]/(ABS control)] × 100(1)

The ABS control was the absorbance of the DPPH radical + water, and the ABS sample was the absorbance of the DPPH radical + tested sample. The results were expressed in Trolox μmol equivalent per gram of sample (μmol/g).

The determination of the antioxidant capacity using ABTS^•+^ radical was performed as previously described [39]. The ABTS radical cation (ABTS^•+^) was generated with the reaction of 5 mL of aqueous ABTS solution (7 mM) + 88 μL of potassium persulfate solution (140 mM). The mixture was kept in the dark (16 h, 28 ± 0.5 °C) before use and diluted with ethanol to achieve an absorbance of 0.7 ± 0.02 units at 734 nm using a UV–VIS spectrophotometer (BEL Photonics, Piracicaba, SP, Brazil). Aliquots (50 µL) of the samples were allowed to react with 250 µL of the resulting blue-green ABTS radical solution in the dark. Decreases in absorbance at 734 nm were measured after 6 min. Controls were prepared with water to replace a sample. For the “blank”, only the extracting solution (300 µL) was used. The antioxidant capacity was calculated using a 2 mM Trolox standard curve (10–2000 µM), and the inhibition percentages were determined with the equation [41]:

ABTS radical scavenging capacity (%) = [(ABS control − ABS sample)]/(ABS control)] × 100(2)

The ABS control was the absorbance of the ABTS radical + water, and the ABS sample was the absorbance of the ABTS radical + tested sample. The results were expressed in Trolox μmol equivalent per gram of sample (μmol/g).

The antioxidant capacity determined by FRAP was measured according to a previously described procedure [33]. Acetate buffer (0.3 M, pH 3.6), 2,4,6-tris(2-pyridyl)-s-triazine (TPTZ) solution (10 mM), and ferric chloride solution (20 mM) were combined to create the FRAP reagent in a ratio of 100:10:10. Aliquots of the extracts in the amount of 20 µL were mixed with 30 µL of distilled water and 250 µL of the reagent. After 30 min at 37 ± 0.5 °C, the mixture absorbance was measured at 595 nm. The “blank” solution was the FRAP reagent itself. The standard curve was built using solutions with known ferrous sulfate concentrations (50–2000 μM). The results were expressed in µmol of ferrous sulfate equivalents per g of sample (µmol FeSO_4_/g).

### 2.6. Statistical Analysis

The experiments were performed in triplicate in three independent repetitions, and the results were expressed as the average ± standard deviation. The Kolmogorov–Smirnov normality test determined the normal distribution of the data. Data were submitted to the Student’s *t*-test or analysis of variance (one-way ANOVA), followed by Tukey’s test. A *p*-value of ≤0.05 was considered statistically significant. The relationship between samples and tested parameters was determined using principal component analysis (PCA). The Pearson’s correlation coefficient (R) and *p*-value were calculated to show the correlations on a heat map. A *p*-value of ≤0.05 was considered statistically significant. GraphPad Prism 7.0 software (GraphPad Software, La Jolla, CA, USA) and R software (Version 2.15.3, Ross Ihaka and Robert Gentleman, University of Auckland, Auckland, New Zealand) were used to run the statistical analysis.

## 3. Results

### 3.1. Changes in Relative Abundance of Intestinal Bacterial Populations during In Vitro Fecal Fermentation

Differences in the relative abundance of the measured intestinal bacterial populations varied with the examined fermentation medium and fecal fermentation period (Table 1). The relative abundance of *Lactobacillus* spp./*Enterococcus* spp. increased (*p* ≤ 0.05) in the different media during the 48 h of fermentation. The highest (*p* ≤ 0.05) relative abundances of *Lactobacillus* spp./*Enterococcus* spp. at 24 h of fermentation were found in the media with ALA5 (7.0 ± 0.36%) and GLA5 (6.93 ± 0.31%). However, the relative abundance of *Lactobacillus* spp./*Enterococcus* spp. at 48 h of fermentation had a two- to three-fold increase (*p* ≤ 0.05) in the media with FOS (12.18 ± 0.39%), GL10 (7.90 ± 0.28%), GLA5 (6.40 ± 0.42%), and AL10 (6.10 ± 0.34%) compared to time zero.

The highest (*p* ≤ 0.05) relative abundances of *Bifidobacterium* spp. at 24 and 48 h of fermentation were found in the media with ALA5 (4.15 ± 0.25–9.2 ± 0.56%) and FOS (4.09 ± 0.18–9.35 ± 0.68%), followed by media with GLA5 (2.86 ± 0.22–7.65 ± 0.47%), GL10 (1.12 ± 0.18–6.62 ± 0.44%), and AL10 (3.38 ± 0.28–4.45 ± 0.51%). The relative abundance of *Bifidobacterium* spp. decreased (*p* ≤ 0.05) in NC at 48 h of fermentation.

The highest (*p* ≤ 0.05) relative abundances of *R. albus*/*R. flavefaciens* at 24 and 48 h of fermentation were found in the medium with ALA5 (6.96 ± 0.39–12.79 ± 0.88%). The relative abundances of *R. albus*/*R. flavefaciens* did not differ (*p* > 0.05) in the media with FOS (9.75 ± 0.63%), GLA5 (9.68 ± 0.74%), and GL10 (9.14 ± 0.65%) at 48 h of fermentation, while the lowest relative abundance (4.08 ± 0.52%) (*p* > 0.05) was found in the medium with AL10. The relative abundance of *R. albus*/*R. flavefaciens* decreased (*p* ≤ 0.05) in NC during the 48 h of fermentation.

The relative abundance of *Bacteroides* spp./*Prevotella* spp. decreased (*p* ≤ 0.05) in the media with AL10 (1.62 ± 0.28%) and GLA5 (2.33 ± 0.37%) at 24 h of fermentation. The relative abundance of *Bacteroides* spp./*Prevotella* spp. increased (*p* ≤ 0.05) in the different media at 48 h of fermentation compared to time zero, except for the medium with GLA5. The medium with GL10 (0.42 ± 0.15–0.67 ± 0.13%) had the lowest (*p* ≤ 0.05) relative abundance of *Bacteroides* spp./*Prevotella* spp. during the 48 h of fermentation.

The lowest (*p* ≤ 0.05) relative abundances of *C. histolyticum* at 24 and 48 h of fermentation were found in the media with AL10 (1.92 ± 0.28–1.16 ± 0.16%) and GL10 (1.85 ± 0.31–0.88 ± 0.16%). The relative abundance of *C. histolyticum* was lower (*p* ≤ 0.05) in all the examined media at 48 h of fermentation compared to time zero, except for NC. The medium with ALA5 had a three-fold decrease in the relative abundance of *C. histolyticum* (10.66 ± 0.98–3.33 ± 0.42%) at 48 h of fermentation compared to time zero.

The relative abundance of *E. rectale*/*C. coccoides* decreased (*p* ≤ 0.05) in the media with FOS (7.0 ± 0.48–5.60 ± 0.32%) and GL10 (1.47 ± 0.29–0.30 ± 0.11%) at 24 and 48 h of fermentation, while it did not change (*p* > 0.05) in the medium with ALA5 and NC. The highest (*p* ≤ 0.05) relative abundances of *E. rectale*/*C. coccoides* were found in the medium with GLA5 (12.71 ± 0.89–10.20 ± 0.76%) at 24 and 48 h of fermentation. The medium with AL10 increased (*p* ≤ 0.05) the relative abundance of *E. rectale*/*C. coccoides* during the 48 h of fermentation.

### 3.2. Microbial Metabolic Activity during In Vitro Fecal Fermentation

Acerola and guava fruit co-products stimulated microbial metabolic activity through decreased pH values, sugar consumption, short-chain fatty acid production, phenolic compound and metabolic profile alteration, and high antioxidant capacity during fecal fermentation. The pH values decreased (*p* ≤ 0.05) in the medium with AL10, ALA5, GL10, GLA5, and FOS during the 48 h of fecal fermentation (Table 2). The lowest pH value (*p* ≤ 0.05) at 24 h of fermentation was found in the medium with AL10 (3.38 ± 0.03), followed by the medium with FOS (3.56 ± 0.01). The highest (*p* ≤ 0.05) pH value was found in NC (5.63 ± 0.02) at 48 h of fermentation, while the lowest pH value was found in the medium with FOS (2.60 ± 0.02). The pH values were low but did not differ in the media with AL10, ALA5, GL10, and GLA5 (3.12 ± 0.01 to 3.17 ± 0.05) (*p* > 0.05) at 48 h of fermentation. The contents of sugars (glucose, fructose, maltose, and rhamnose) decreased (*p* ≤ 0.05) in the media with AL10, ALA5, GL10, GLA5, and FOS during the 48 h of fermentation, while sugars were not detected in NC. Rhamnose was found only in the media with AL10, ALA5, GL10, and GLA5. Glucose, fructose, and maltose (0.15 ± 0.01–3.59 ± 0.04 g/L) were detected only in the medium with FOS at 48 h of fermentation. 

The lactic acid contents increased (*p* ≤ 0.05) in the media with AL10, ALA5, GL10, GLA5, and FOS at 24 h of fermentation. However, lactic acid was detected in the medium with FOS (7.83 ± 0.05 g/L) at 48 h of fermentation. The contents of acetic and butyric acids increased (*p* ≤ 0.05) during the 48 h of fermentation in the media with AL10, ALA5, GL10, GLA5, and FOS, which were higher than (*p* ≤ 0.05) in NC. The media with AL10 and GL10 had higher contents of butyric acid (2.03 ± 0.01 g/L–2.11 ± 0.06) at 48 h of fermentation. The medium with FOS had the highest (*p* ≤ 0.05) content of acetic acid (2.13 ± 0.02 g/L) after 48 h of fermentation. Propionic acid was detected in all the examined fermentation media; however, the contents of propionic acid decreased (*p* ≤ 0.05) during 48 h of fermentation. 

**Figure 1:**
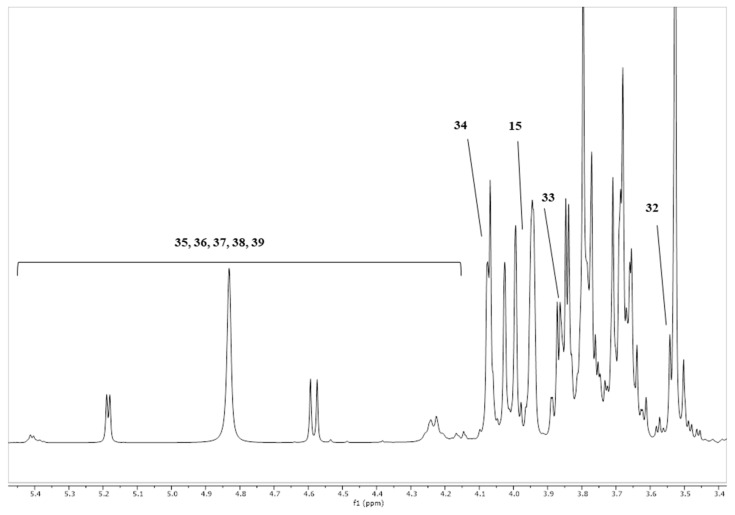

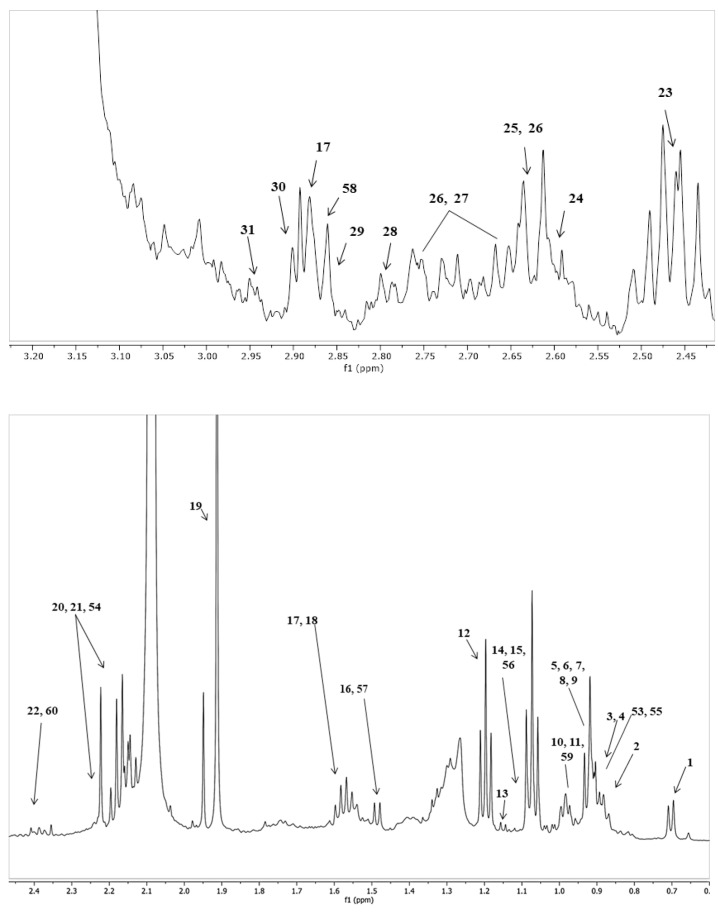

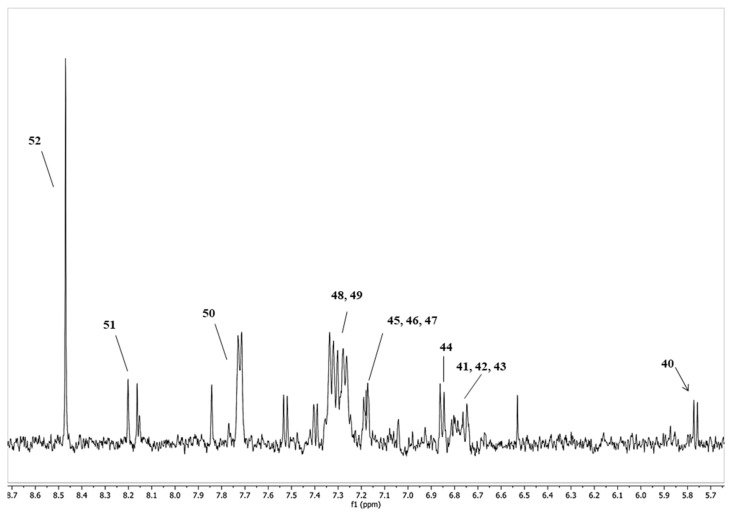
Representative ^1^H NMR spectra of the media with fermented acerola and guava fruit co-products digested at time zero and 48 h of in vitro fecal fermentation analyzed by ^1^H NMR. 1: biliary salts; 2: 2-methylbutyrate; 3: valerate; 4: n-butyrate; 5: leucine; 6: isoleucine; 7: valine; 8: propionate/propionic acid; 9: isobutyrate/butyric acid; 10: 3-methyl-2-oxoisovalerate; 11: 2-oxoisovalerate; 12: ethanol; 13: 3-hydroxybutyrate; 14: threonine; 15: lactate/lactic acid; 16: alanine; 17: lysine; 18: ornithine; 19: acetate/acetic acid; 20: proline; 21: glutamate; 22: 5-aminopentanoate; 23: succinate/succinic acid; 24: methylamine; 25: methionine; 26: citrate/citric acid; 27: aspartate; 28: asparagine; 29: trimethylamine; 30: putrescine; 31: malonate; 32: glycine; 33: fructose; 34: dihydroxyacetone; 35: α-xylose; 36: β-xylose; 37: β-glucose; 38: α-glucose; 39: β-galactose; 40: UDP-glucuronate; 41: homovanillate; 42: 3-hydroxyphenylacetate; 43: p-cresol; 44: tyrosine; 45: 5-aminosalicylate; 46: phenylalanine; 47: uracil; 48: N-acetyl-5-aminosalicylate; 49: phenylacetate; 50: tryptophan; 51: hypoxanthine; 52: formate/formic acid; 53: caprylate; 54: isocaproate; 55: isovalerate; 56: 3-hydroxyisovalerate; 57: total lipids; 58: gamma-aminobutyric acid (GABA); 59: ketoisovalerate; 60: acetone.

The RMN Global metabolic profiling identified 60 different chemical compounds in the media with AL10, ALA5, GL10, GLA5, FOS, and NC at time zero and 48 h of fecal fermentation (Figure 1 and Supplementary Materials Table S2). Alanine, lysine, ornithine, methylamine, trimethylamine, putrescine, malonate, and gamma-aminobutyric acid (GABA), as well as several organic acids (acetic, succinic, and formic acids), were identified in all the examined media at time zero and 48 h of fecal fermentation (Figure 1). However, some compounds, such as leucine, isoleucine, valine, methionine, tryptophan, hypoxanthine, and 3-hydroxy isovalerate, detected at the beginning of the fermentation, lost signal intensity at 48 h of fermentation in the media with AL10, ALA5, GL10, and GLA5. Some compounds were detected only after 48 h of fermentation, notably threonine and 3-hydroxyisovalerate. Sugars (fructose, α-xylose, β-xylose, β-glucose, α-glucose, and D-galactose) had a reduction or loss of signals (non-detection) at 48 h of fermentation.

### 3.3. Changes in Phenolic Compounds and Antioxidant Capacity during In Vitro Fecal Fermentation

Ten distinct phenolic compounds belonging to three distinct classes were identified in the media with AL10, ALA5, GL10, and GLA5 during the 48 h of fecal fermentation: two phenolic acids, six flavonoids, and two flavonols (Table 3). Gallic acid, procyanidin A2, procyanidin B1, procyanidin B2, and epigallocatechin gallate were the most prevalent phenolic compounds in all the examined media during fermentation (Table 3). The contents of procyanidin A2, procyanidin B1, and procyanidin B2 were reduced (*p* ≤ 0.05) at 24 or 48 h of fermentation, while the contents of epigallocatechin gallate increased (*p* ≤ 0.05) at 24 or 48 h of fermentation. Gallic acid was detected at 24 h of fermentation in all the examined media, and its contents decreased (*p* ≤ 0.05) at 48 h of fermentation. 

The antioxidant capacity was increased (*p* ≤ 0.05) or maintained (*p* > 0.05) in all the analyzed media during 48 h of fecal fermentation, except for the media with GLA10 and GLA5, in which the antioxidant capacity decreased when measured using the FRAP method (Table 3). The media with ALA5 and GLA5 (28.79 ± 0.22 and 28.50 ± 0.15 μmol/g, respectively) had higher antioxidant capacity (*p* ≤ 0.05) at 48 h of fermentation compared to time zero when measured with the ABTS method.

### 3.4. Chemometric Analysis

**Figure 2:**
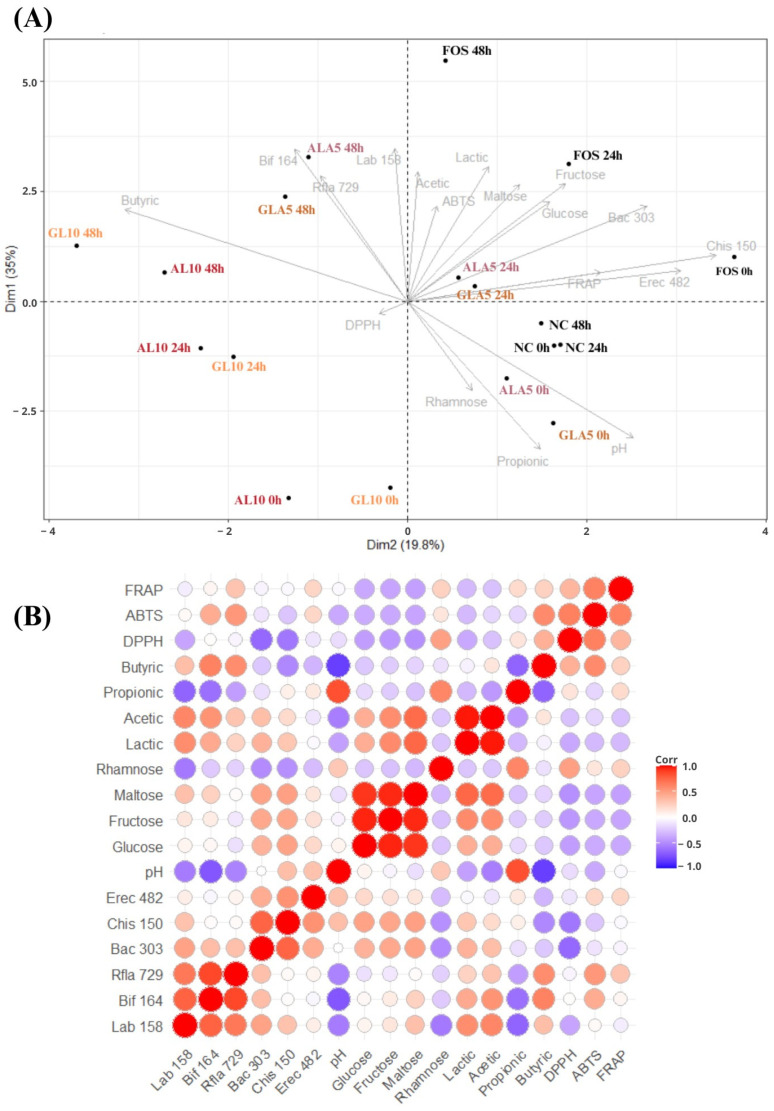
(**A**) Principal component analysis (PCA) run in media with fermented acerola and guava fruit co-products (AL10, ALA5, GL10, and GLA5), fructooligosaccharides (FOS), and negative control (NC; without fermentable substrate) at time zero (baseline) and 24 and 48 h of in vitro fecal fermentation (variables: relative abundance of distinct bacterial groups, contents of sugars and organic acids, pH values, and antioxidant capacity). (**B**) Heat map with correlation coefficients indicates the associations between the relative abundance of distinct bacterial groups, contents of sugars and organic acids, pH values, and antioxidant capacity.

The PCA results (Figure 2A) located the media with AL10, ALA5, GL10, GLA5, and FOS at 48 h of fecal fermentation in the upper quadrant with the higher contents of SCFA and lactic acid; a higher relative abundance of *Lactobacillus* spp./*Enterococcus* spp., *Bifidobacterium* spp., and *R. albus*/*R. flavefaciens*; and the highest antioxidant capacity measured with the ABTS method. NC at 24 and 48 h of fermentation was in the lower right quadrant, with a higher relative abundance of *C. histolyticum* and *E. rectale*/*C. coccoides*. 

The Pearson’s correlation test (Figure 2B) showed that *Lactobacillus* spp./*Enterococcus* spp., *Bifidobacterium* spp., and *R. albus*/*R. flavefaciens* correlated positively (*p* < 0.001) with lactic, acetic, and butyric acids and antioxidant capacity measured with the ABTS method while correlating negatively (*p* < 0.001) with pH values. *Bacteroides* spp./*Prevotella* spp., *C. histolyticum*, and *E. rectale*/*C. coccoides* negatively correlated (*p* < 0.001) with antioxidant capacity measured with the ABTS and FRAP methods while correlating positively (*p* < 0.001) with pH values and sugars (glucose, fructose, and maltose).

## 4. Discussion

Acerola and guava co-products fermented with probiotic *L. acidophilus* and *L. paracasei* promoted increases in the relative abundance of *Lactobacillus* spp./*Enterococcus* spp. and *Bifidobacterium* spp. populations during 48 h of fecal fermentation, which indicates high amounts of non-digestible compounds in these materials, such as insoluble and soluble fibers and phenolic compounds used as substrates by these intestinal microorganisms [4]. *Lactobacillus* spp. and *Bifidobacterium* spp. are the major microbial targets linked to human intestinal health, being widely used as probiotics and determinants of the prebiotic effects of various foods, while some *Enterococcus* species found in the healthy intestinal microbiota are considered potentially probiotics [33,42]. The highest relative abundance of *Lactobacillus* spp./*Enterococcus* spp. found in the medium with FOS confirms its use as a recognized prebiotic [11]. The increase in the relative abundance of *Lactobacillus* spp./*Enterococcus* spp. in the media with AL10, ALA5, GL10, and GLA5 during fecal fermentation could be partially linked to the presence of the fermentative probiotic strains, which kept viable cell counts of >9 log CFU/g in the tested fermented fruit co-products (Table 1). Although the beneficial effects of probiotics are strain- and dose-dependent, these viable cell counts are greater than the minimum of 6 log CFU/g commonly reported to reach the claimed beneficial effects for consumers [43]. 

*R. albus*/*R. flavefaciens* are emerging beneficial intestinal bacterial species that are more abundant in healthy individuals, acting as cellulolytic bacteria and playing an important role in fiber breakdown in the human intestine [44,45]. The medium with ALA5 had a higher relative abundance of *R. albus*/*R. flavefaciens* than FOS. The increase in the relative abundance of *R. albus*/*R. flavefaciens* during fecal fermentation could be related to the presence of probiotic lactobacilli in the media with AL10, ALA5, GL10, and GLA5, as these probiotics could make the environment more favorable for *Ruminococcus* species [46]. Increased *Lactobacillus* spp., *Bifidobacterium* spp., and *Ruminococcus* spp. populations help to ameliorate intestinal diseases (e.g., inflammatory bowel disease and irritable bowel syndrome), metabolic (obesity, diabetes, and cardiovascular disease), and immune-related diseases, in addition to acting in the gut–brain axis with neuroprotective and antidepressant effects [45,47,48,49].

*Bacteroides* spp./*Prevotella* spp. can break down high-molecular-weight polysaccharides, such as insoluble and soluble fiber, in the fermented acerola and guava co-products to produce acetic acid and mainly propionic acid [50]. Variations in the population of *Bacteroides* spp./*Prevotella* spp. could be related to competition with other microorganisms for environmental nutrients because these bacteria do not have selective substrates to consume [16]. The medium with ALA5 showed the behavior of this bacterial group, like the medium with FOS, during fecal fermentation. The media with AL10, ALA5, GL10, and GLA5 maintained or reduced the relative abundance of *Bacteroides* spp./*Prevotella* spp. until 24 h of fecal fermentation, which could be a positive effect because increased populations of these commonly opportunistic bacteria are linked to undesirable outcomes in intestinal health, while less abundant populations are typically considered beneficial to intestinal health [46,51]. 

*C. histolyticum* is a well-known enteric pathogen, and a decreased relative abundance of this bacterium during fecal fermentation could reinforce the selective stimulatory effects of the fermented acerola and guava co-products on beneficial groups forming the intestinal microbiota [52]. The decrease in the relative abundance of *C. histolyticum* could be due to a decrease in pH values and an increase in amounts of specific phenolic compounds (e.g., gallic acid, procyanidin B2, and epigallocatechin gallate) during fecal fermentation, which are limiting factors for *C. histolyticum* growth [53]. The increase in relative abundance of *C. histolyticum* in NC at 24 h of fermentation could be related to the availability of food remains in the fecal inoculum but without the capacity to sustain this viability and growth up to 48 h of fermentation.

The relative abundance of *E. rectale*/*C. coccoides* varied in the media with acerola and guava during fecal fermentation, which could be related to how this microorganism metabolizes co-products in the presence of each probiotic strain. *E. rectale*/*C. coccoides* belong to the *Clostridium* cluster XIVa, which are important regulators of intestinal homeostasis. However, to evaluate potential prebiotic effects, these species were not included as beneficial due to safety concerns and probiotic efficacy limitations [54]. These results showing changes in fecal bacterial populations contribute to the development of novel synbiotic ingredients from recycled fruit processing co-products because they strengthen the evidence of their use as functional foods and promote a new route for using these co-products.

The sugars, such as glucose, fructose, maltose, and rhamnose, were metabolized in the media with AL10, ALA5, GL10, GLA5, and FOS during fecal fermentation, indicating intense fermentative microbial metabolic activity [31,55]. This activity resulted in the production of organic acids, leading to a gradual decrease in pH over time (Table 2). These conditions could favor increased absorption of certain nutrients while inhibiting the growth of pathogenic microorganisms [56]. The increase in the rhamnose contents at 48 h of fermentation could indicate the ability of the fecal microbiota to degrade rhamnose-containing polysaccharides (e.g., pectin) present in acerola and guava co-products to monosaccharides [15].

SCFA are volatile fatty acids produced in the large intestine due to the fermentation of food components by the intestinal microbiota and are directly linked to general intestinal health [56]. Acetic, propionic, and butyric acids are the most common SCFAs generated by the intestinal microbiota [57]. In this study, the simulation of fecal fermentation showed the capacity of AL10, ALA5, GL10, GLA5, and FOS to reduce the pH, especially through acetic and butyric acid production (Table 2), as also observed in Pearson’s correlation test, where these acids correlated negatively with pH values, supporting the synbiotic potential of fermented co-products. Butyric acid, belonging to the group of monocarboxylic acids, is predominantly produced by bacteria, such as *Lactobacillus* spp., *Ruminococcus* spp., and *Clostridium* spp., during the fermentation of non-digestible carbohydrates, proteins, and lactic acid [58]. Although *Clostridium* spp. is a butyric acid producer, this genus is associated with producing some toxic metabolites often considered detrimental to intestinal health [12]. At the end of the 48 h of fecal fermentation, butyric acid contents had more than a 2-fold increase in media with probiotic fermented acerola and guava co-products compared to NC and FOS, which could be linked to variations in the relative abundance of *E. rectale*/*C. cocoides*, as observed by a negative correlation in Pearson’s test between butyric acid and *E. rectale*/*C. cocoides* abundance. These metabolic changes could contribute to the potential health benefits of the fermented co-products at the intestinal level. Butyric acid is the primary energy source for colonocytes, regulating gene expression and promoting anti-inflammatory and anticancer effects [59,60,61]. Acetic acid is a carboxylic acid associated with the metabolism of several bacterial groups, including *Lactobacillus* spp., *Bifidobacterium* spp., *Ruminococcus* spp., and *Bacteroides* spp. [62], which agrees with the results of Pearson’s correlation test showing that these bacterial groups correlated positively with lactic, acetic, and butyric acids. The media with AL10, ALA5, GL10, and GLA5 had increased populations of *Lactobacillus* spp., *Bifidobacterium* spp., and *Ruminococcus* spp. during fecal fermentation, which may be related to the parallel increase in the contents of acetic acid in these media [63], which also corroborates the correlations observed in the Pearson’s correlation test.

The medium with FOS had higher contents of glucose and fructose, probably linked to the higher contents of lactic and acetic acids and lower pH than the other examined fermentation media (Table 2). This probably occurred because more straightforward carbohydrates were more available for metabolization [64] and due to the high relative abundance of *Lactobacillus* spp./*Enterococcus* spp. observed in the medium with FOS [31]. Low contents of propionic acid were found during fecal fermentation in all the examined fermentation media, but the lower contents of this SCFA were found in the media with GLA10 and GLA5, which may be linked to the decreased relative abundance of *Bacteroides* spp./*Prevotella* spp. therein [31,53]. In addition, the metabolism of these bacteria can be influenced by the relatively low pH observed in in vitro assays, directly affecting propionic acid production [65].

Regardless of the fermentation environment, the overall metabolic profiling showed a wide range of chemical compounds commonly detected in human feces [66] in the media with AL10, ALA5, GL10, and GLA5 during fecal fermentation. The media with AL10, ALA5, GL10, and GLA5 had a reduction in the intensity of signals related to saccharide monomers at 48 h of fecal fermentation (Table 2). Consequently, different organic acids, such as succinic, acetic, and formic acids, were mainly identified in these media. These acids (acetic, formic, citric, succinic, and lactic acids) are produced by bacteria forming the large intestine microbiota through the fermentation of non-digestible carbohydrates [56,67], which agrees with the results of the quantification of organic acids and sugars and pH reduction during fecal fermentation and reinforces the synbiotic potential of probiotic fermented co-products.

Several amino acids were identified in the media with AL10, ALA5, GL10, and GLA5 at 48 h of fecal fermentation, including alanine, lysine, ornithine, proline, tyrosine, and threonine. Identifying these amino acids could be due to a predominantly saccharolytic metabolism in most intestinal microorganisms, which inhibits the fermentation of amino acids in favor of carbohydrates [68]. Therefore, the presence of non-digestible carbohydrates in fermentation media may have stimulated bacteria with saccharolytic metabolism to produce organic acids. Despite this, there was still a reduction in the signals of some amino acids, such as isoleucine, valine, leucine, lysine, and methionine, during fecal fermentation. Many of these amino acids can participate in several metabolic pathways involving amino acids, carbohydrates, and nucleotides [69,70]. Threonine could stand out among the amino acids detected at 48 h of fermentation because it is essential in maintaining mucosal integrity and barrier function by supporting mucin secretion [71].

Phenolic compounds and the intestinal microbiota have a mutually beneficial relationship because the intestinal microbiota metabolizes phenolic compounds into absorbable and more accessible derived metabolites, while phenolic compounds can induce increases in the abundance and diversity of commensal bacteria in the intestinal microbiota [72,73]. The decrease in phenolic compounds during fecal fermentation indicates their metabolization by the intestinal microbiota [13]. The contents of phenolic compounds decreased in the media with AL10, ALA5, GL10, and GLA5 during fecal fermentation, with the exception of epigallocatechin gallate, which increased or maintained the content during fecal fermentation. The hydrolysis of other phenolic compounds found in AL10, ALA5, GL10, and GLA5 released during intestinal digestion and fecal fermentation could be related to increased contents of epigallocatechin gallate as well as the detection of gallic acid after 24 h of fecal fermentation [33]. Interestingly, epigallocatechin gallate is associated with selective stimulation of *Lactobacillus* and *Bifidobacterium* in the human colon and may serve as a substrate for beneficial intestinal microbiota [74], as observed by the increase in the relative abundance of these bacterial groups.

The contents of gallic acid, procyanidin A2, and procyanidin B2 decreased (*p* ≤ 0.05) during fecal fermentation, although these compounds were detected until 48 h of fermentation (Table 3). A previous investigation reported the conversion of procyanidins to epicatechin in an in vitro fecal fermentation model [75], agreeing with the results of this study. Procyanidins (free or associated with the cell wall) are poorly bioavailable in the upper part of the intestine and reach the colon to become fermentable substrates for the commensal microbiota. Although the microbial catabolism of procyanidins is far from being completely described, it is known that procyanidins coming from food matrices, such as fruits, tend to be transformed into readily absorbable low-molecular-weight metabolites [76,77,78]. *Lactobacillus* species produce gallate decarboxylase, an enzyme that degrades gallic acid into other compounds, such as oxaloacetate and pyruvate, used in the Krebs cycle [79,80]. Despite the decrease during the fecal fermentation, the presence of gallic acid and other phenolic compounds in the samples is important because they could exert many beneficial effects on human health, such as acting as an antioxidant, anti-inflammatory, antidiabetic, and anticancer agent [81,82].

The antioxidant capacity overall increased or remained in all the examined media during fecal fermentation when measured with the DPPH and ABTS methods (Table 3). However, the antioxidant capacity in the media with GLA10 and GLA5 decreased when measured with the FRAP method. Different chemical properties of compounds, principles, and conditions can lead to differences in results obtained with distinct methods to assess antioxidant capacity [83]. Nevertheless, the DPPH and ABTS methods are considered efficient in evaluating the antioxidant capacity of fecal fermentation media [30]. The reported high antioxidant capacity may be due to the presence of different phenolic compounds in guava and acerola co-products as well as their metabolization by the intestinal microbiota [84]. The modulation of gut microbiota can additionally enhance antioxidant capacity, as evidenced in results from Pearson’s correlation test, where beneficial bacterial populations (*Lactobacillus* spp./*Enterococcus* spp., *Bifidobacterium* spp., and *R. albus*/*R. flavefaciens*) positively correlated with antioxidant capacity, while non-beneficial bacterial populations (*C. histolyticum* and *E. rectale*/*C. coccoides*) negatively correlated with antioxidant capacity. This suggests a potential relationship between gut microbiota composition and antioxidant capacity in the intestinal environment [85]. The presence of gallic acid, epigallocatechin gallate, and procyanidin B2 in the media with ALA5, GL10, and GLA5 at 48 h of fecal fermentation could also contribute to their high antioxidant capacity. 

The beneficial impacts of phenolic compounds on intestinal health are primarily linked to their antioxidant capacity. These effects are particularly important in the large intestine due to the persistent action of dietary-derived oxidants, mutagens, carcinogens, and internally produced reactive oxygen species [85,86]. Metabolites possessing antioxidant properties in the intestinal environment may uphold a reduced redox state, particularly within mucosal cells, with systemic beneficial repercussions due to inhibition of free radical production [86].

A principal component analysis (PCA) (Figure 2A) revealed that all the analyzed media containing probiotic fermented acerola and guava co-products could induce favorable alterations during the 48 h of fecal fermentation, which was overall comparable to the alterations induced by FOS, a well-established prebiotic ingredient. Fermentation with probiotics could improve the functional properties of fruit co-products and the bioaccessibility of nutrients, promoting the production of active bacterial metabolites (e.g., anti-inflammatory and antioxidant metabolites) in the human colon, playing a regulatory role in transepithelial fluid transport, improving the inflammatory status of the mucosa, and modulating visceral sensitivity and intestinal motility [87]. Although in vitro studies to evaluate prebiotic properties using human fecal inoculum have proven to be an effective and easily reproducible methodology on a laboratory scale, future in vivo studies are necessary to confirm the findings of this investigation.

## 5. Conclusions

The fecal fermentation of AL10, ALA5, GL10, and GLA5 with probiotic lactobacilli increased the relative abundance of bacterial populations commonly reported to benefit intestinal health, in addition to decreasing or maintaining the relative abundance of non-beneficial bacterial populations. Sugars and phenolic compounds present in media with fermented acerola and guava fruit co-products were extensively metabolized by the intestinal microbiota during fecal fermentation. This process resulted in reduced pH values, increased production of SCFA, an altered metabolic profile, and maintenance of antioxidant capacity, which are effects compatible with prebiotic compounds, indicating the potential of the tested fermented co-products to improve intestinal health. The elaboration of fermented fruit co-products may prove to be a potential strategy to increase their use and valorization as novel functionalized synbiotic circular ingredients with low cost and to develop value-added foods and dietary supplements of non-dairy origin to meet the demands of different consumers.

## Figures and Tables

**Table 1:** Relative abundance (% average ± standard deviation; n = 3) of different intestinal bacterial groups in the media with digested fermented acerola and guava fruit co-products (AL10, ALA5, GL10, and GLA5), fructooligosaccharides (FOS), and negative control (NC; without fermentable substrate) at time zero and 24 and 48 h of in vitro fecal fermentation.

Bacterial Groups	Fermentation Medium	Fermentation Period		
		0 h	24 h	48 h
*Lactobacillus* spp./*Enterococcus* spp.	NC	5.89 ± 0.31 ^Ba^	6.23 ± 0.35 ^ABb^	6.68 ± 0.41 ^Ac^
	FOS	4.81 ± 0.16 ^Cb^	5.76 ± 0.28 ^Bb^	12.18 ± 0.39 ^Aa^
	AL10	3.06 ± 0.22 ^Cc^	3.88 ± 0.17 ^Bc^	6.10 ± 0.34 ^Ac^
	ALA5	6.20 ± 0.28 ^Ca^	7.00 ± 0.36 ^Ba^	7.84 ± 0.26 ^Ab^
	GL10	2.70 ± 0.14 ^Cc^	4.34 ± 0.25 ^Bc^	7.90 ± 0.28 ^Ab^
	GLA5	2.65 ± 0.20 ^Bc^	6.93 ± 0.31 ^Aa^	6.40 ± 0.42 ^Ac^
*Bifidobacterium* spp.	NC	2.43 ± 0.22 ^Abc^	2.70 ± 0.26 ^Ac^	1.84 ± 0.16 ^Be^
	FOS	2.71 ± 0.20 ^Cb^	4.09 ± 0.18 ^Ba^	9.35 ± 0.68 ^Aa^
	AL10	1.53 ± 0.19 ^Cd^	3.38 ± 0.28 ^Bb^	4.45 ± 0.51 ^Ad^
	ALA5	3.60 ± 0.27 ^Ca^	4.15 ± 0.25 ^Ba^	9.20 ± 0.56 ^Aa^
	GL10	1.44 ± 0.16 ^Bd^	1.12 ± 0.18 ^Bd^	6.62 ± 0.44 ^Ac^
	GLA5	2.14 ± 0.18 ^Cc^	2.86 ± 0.22 ^Bc^	7.65 ± 0.47 ^Ab^
*Ruminococcus albus/R. flavefaciens*	NC	5.38 ± 0.27 ^Aa^	5.07 ± 0.24 ^Ab^	1.77 ± 0.20 ^Bd^
	FOS	3.0 ± 0.21 ^Cb^	4.05 ± 0.29 ^Bc^	9.75 ± 0.63 ^Ab^
	AL10	1.57 ± 0.18 ^Bd^	1.73 ± 0.12 ^Bd^	4.08 ± 0.52 ^Ac^
	ALA5	5.39 ± 0.30 ^Ca^	6.96 ± 0.39 ^Ba^	12.79 ± 0.88 ^Aa^
	GL10	2.03 ± 0.26 ^Cc^	4.79 ± 0.23 ^Bbc^	9.14 ± 0.65 ^Ab^
	GLA5	4.98 ± 0.39 ^Ba^	4.26 ± 0.35 ^Bc^	9.68 ± 0.74 ^Ab^
*Bacteroides* spp./*Prevotella* spp.	NC	6.33 ± 0.51 ^Bb^	9.50 ± 0.64 ^Aa^	8.40 ± 0.55 ^Ab^
	FOS	8.14 ± 0.47 ^Ba^	9.61 ± 0.69 ^Aa^	9.23 ± 0.62 ^ABab^
	AL10	2.26 ± 0.19 ^Bd^	1.62 ± 0.28 ^Cc^	3.11 ± 0.33 ^Ad^
	ALA5	8.83 ± 0.46 ^Aa^	9.16 ± 0.71 ^Aa^	9.83 ± 0.60 ^Aa^
	GL10	0.42 ± 0.15 ^Ae^	0.43 ± 0.13 ^Ad^	0.67 ± 0.13 ^Aa^
	GLA5	5.0 ± 0.32 ^Ac^	2.33 ± 0.37 ^Bb^	4.33 ± 0.38 ^Ac^
*Clostridium histolyticum*	NC	5.71 ± 0.47 ^Bc^	9.73 ± 0.64 ^Aa^	6.21 ± 0.29 ^Bb^
	FOS	9.11 ± 0.52 ^Ab^	6.20 ± 0.51 ^Bb^	7.66 ± 0.45 ^Ca^
	AL10	1.81 ± 0.14 ^Ae^	1.92 ± 0.28 ^Ac^	1.16 ± 0.16 ^Bd^
	ALA5	10.66 ± 0.98 ^Aa^	6.16 ± 0.57 ^Bb^	3.33 ± 0.42 ^Cc^
	GL10	1.89 ± 0.22 ^Ae^	1.85 ± 0.31 ^Ac^	0.88 ± 0.16 ^Bd^
	GLA5	4.60 ± 0.37 ^Ad^	5.60 ± 0.68 ^Ab^	3.70 ± 0.27 ^Bc^
*Eubacterium rectale/C. coccoides*	NC	9.83 ± 0.47 ^Aa^	8.96 ± 0.44 ^ABb^	8.20 ± 0.49 ^Bb^
	FOS	9.60 ± 0.59 ^Aa^	7.0 ± 0.48 ^Bc^	5.60 ± 0.32 ^Cd^
	AL10	1.92 ± 0.14 ^Cd^	2.85 ± 0.26 ^Bd^	3.89 ± 0.28 ^Ae^
	ALA5	5.83 ± 0.40 ^Ab^	6.33 ± 0.35 ^Ac^	6.66 ± 0.49 ^Ac^
	GL10	4.71 ± 0.52 ^Ac^	1.47 ± 0.29 ^Be^	0.30 ± 0.11 ^Ca^
	GLA5	9.33 ± 0.81 ^Ba^	12.71 ± 0.89 ^Aa^	10.20 ± 0.76 ^Aba^

AL10: acerola + *L. paracasei* L10; ALA5: acerola + *L. acidophilus* LA-05; GL10: guava + *L. paracasei* L10; GLA5: guava + *L. acidophilus* LA-05. A–C: different superscript capital letters in the same row for the same fermentation medium denote differences (*p* ≤ 0.05) based on Tukey’s test; a–e: different superscript small letters in the same column at the same time interval and bacterial group denote differences (*p* ≤ 0.05) based on Tukey’s test.

**Table 2:** pH values, contents of sugars, lactic acid, and short-chain fatty acids (SCFA) (g/L) in media with fermented acerola and guava fruit co-products (AL10, ALA5, GL10, and GLA5), fructooligosaccharides (FOS), and negative control (NC; without fermentable substrate) at time zero (baseline) and 24 and 48 h of in vitro fecal fermentation.

Parameters	Samples	Fermentation Period		
		0 h	24 h	48 h
pH values	NC	7.07 ± 0.01 ^Aa^	6.81 ± 0.02 ^Ba^	5.63 ± 0.02 ^Ca^
	FOS	7.06 ± 0.01 ^Aa^	3.56 ± 0.01 ^Be^	2.60 ± 0.02 ^Cd^
	AL10	7.00 ± 0.02 ^Ab^	3.38 ± 0.03 ^Bf^	3.17 ± 0.01 ^Cb^
	ALA5	6.96 ± 0.00 ^Ac^	4.33 ± 0.02 ^Bc^	3.13 ± 0.00 ^Cc^
	GL10	6.93 ± 0.01 ^Ad^	4.24 ± 0.00 ^Bd^	3.12 ± 0.01 ^Cc^
	GLA5	6.93 ± 0.01 ^Ad^	4.99 ± 0.00 ^Bb^	3.12 ± 0.01 ^Cc^
*Sugars (g/L)*				
Glucose	NC	<LOD	<LOD	<LOD
	FOS	8.47 ± 0.03 ^Aa^	5.31 ± 0.04 ^Ba^	2.94 ± 0.01 ^Ca^
	AL10	0.43 ± 0.04 ^Ac^	<LOD	<LOD
	ALA5	0.31 ± 0.04 ^Ad^	<LOD	<LOD
	GL10	0.48 ± 0.01 ^Ab^	<LOD	<LOD
	GLA5	0.47 ± 0.01 ^Ab^	<LOD	<LOD
Fructose	NC	<LOD	<LOD	<LOD
	FOS	7.28 ± 0.01 ^Ba^	7.55 ± 0.03 ^Aa^	3.59 ± 0.04 ^Ca^
	AL10	0.18 ± 0.01 ^Ac^	<LOD	<LOD
	ALA5	0.12 ± 0.05 ^Ad^	<LOD	<LOD
	GL10	0.25 ± 0.04 ^Ab^	0.07 ± 0.01 ^Bb^	<LOD
	GLA5	0.24 ± 0.02 ^Ab^	<LOD	<LOD
Maltose	NC	<LOD	<LOD	<LOD
	FOS	0.16 ± 0.04 ^Aa^	0.15 ± 0.03 ^Aa^	0.15 ± 0.01 ^Aa^
	AL10	0.02 ± 0.01 ^Ab^	<LOD	<LOD
	ALA5	0.02 ± 0.01 ^Ab^	<LOD	<LOD
	GL10	<LOD	<LOD	<LOD
	GLA5	<LOD	<LOD	<LOD
Rhamnose	NC	<LOD	<LOD	<LOD
	FOS	<LOD	<LOD	<LOD
	AL10	0.40 ± 0.01 ^Ac^	0.22 ± 0.04 ^Ba^	0.17 ± 0.01 ^Bb^
	ALA5	0.13 ± 0.04 ^Bd^	<LOD	0.23 ± 0.04 ^Aa^
	GL10	0.84 ± 0.02 ^Aa^	0.15 ± 0.03 ^Cb^	0.28 ± 0.04 ^Ba^
	GLA5	0.61 ± 0.02 ^Ab^	0.06 ± 0.01 ^Cc^	0.16 ± 0.01 ^Bb^
*Acids (g/L)*				
Lactic	NC	<LOD	<LOD	<LOD
	FOS	<LOD	5.35 ± 0.05 ^Ba^	7.83 ± 0.05 ^Aa^
	AL10	0.13 ± 0.01 ^Ba^	0.17 ± 0.01 ^Ad^	<LOD
	ALA5	0.13 ± 0.01 ^Ba^	0.24 ± 0.02 ^Ac^	<LOD
	GL10	0.11 ± 0.02 ^Ba^	0.42 ± 0.04 ^Ab^	<LOD
	GLA5	<LOD	0.39 ± 0.02 ^Ab^	<LOD
Acetic	NC	0.22 ± 0.02 ^Bc^	0.25 ± 0.00 ^Ac^	0.21 ± 0.00 ^Bd^
	FOS	0.36 ± 0.03 ^Ca^	1.59 ± 0.01 ^Ba^	2.13 ± 0.02 ^Aa^
	AL10	0.30 ± 0.05 ^Ca^	0.45 ± 0.06 ^Bb^	0.58 ± 0.01 ^Ab^
	ALA5	0.32 ± 0.03 ^Ba^	0.39 ± 0.05 ^Bb^	0.49 ± 0.01 ^Ac^
	GL10	0.34 ± 0.02 ^Ca^	0.45 ± 0.01 ^Ab^	0.48 ± 0.05 ^Bc^
	GLA5	0.26 ± 0.01 ^Bb^	0.41 ± 0.01 ^Ab^	0.51 ± 0.08 ^Ac^
Propionic	NC	1.09 ± 0.00 ^Ae^	0.99 ± 0.02 ^Ba^	0.40 ± 0.00 ^Ca^
	FOS	0.68 ± 0.06 ^Af^	0.39 ± 0.00 ^Bd^	0.31 ± 0.01 ^Cb^
	AL10	1.32 ± 0.02 ^Ac^	0.62 ± 0.01 ^Bb^	0.34 ± 0.04 ^Cb^
	ALA5	1.16 ± 0.02 ^Ad^	0.65 ± 0.03 ^Bb^	0.41 ± 0.06 ^Ca^
	GL10	1.40 ± 0.01 ^Ab^	0.61 ± 0.02 ^Bb^	0.20 ± 0.01 ^Cd^
	GLA5	1.74 ± 0.08 ^Aa^	0.56 ± 0.02 ^Bc^	0.24 ± 0.00 ^Cc^
Butyric	NC	0.23 ± 0.00 ^Bc^	0.97 ± 0.01 ^Ad^	<LOD
	FOS	0.29 ± 0.01 ^Cb^	0.68 ± 0.00 ^Be^	0.79 ± 0.00 ^Ad^
	AL10	0.23 ± 0.00 ^Cc^	1.34 ± 0.01 ^Ba^	2.03 ± 0.01 ^Ab^
	ALA5	0.28 ± 0.03 ^Cb^	1.27 ± 0.00 ^Bb^	1.86 ± 0.05 ^Ac^
	GL10	0.22 ± 0.02 ^Cc^	1.09 ± 0.00 ^Bc^	2.11 ± 0.06 ^Aa^
	GLA5	0.33 ± 0.01 ^Ca^	1.08 ± 0.05 ^Bc^	1.77 ± 0.09 ^Ac^

AL10: acerola + *L. paracasei* L10; ALA5: acerola + *L. acidophilus* LA-05; GL10: guava + *L. paracasei* L10; GLA5: guava + *L. acidophilus* LA-05. A–C: different superscript capital letters in the same row denote differences (*p* ≤ 0.05) based on the Student’s *t*-test or Tukey’s test; a–f: different superscript small letters in the same column for the same time interval and measured parameter denote differences (*p* ≤ 0.05) based on the Student’s *t*-test or Tukey’s test. <LOD: below the limit of detection (LOD was 0.02 g/L for glucose, 0.05 g/L for fructose, 0.02 g/L for lactic acid, 0.007 g/L for malic acid, 0.001 g/L for acetic acid, 0.024 g/L for propionic acid, and 0.02 g/L for butyric acid).

**Table 3:** Contents of phenolic compounds (mg/L) and antioxidant capacity (%) (average ± standard deviation; n = 3) in media with fermented acerola and guava fruit co-products (AL10, ALA5, GL10, and GLA5), fructooligosaccharides (FOS), and negative control (NC; without fermentable substrate) at time zero (baseline) and 24 and 48 h of in vitro fecal fermentation.

Parameters	Samples	Fermentation Period		
		0 h	24 h	48 h
*Phenolic acids*				
Gallic acid	AL10	<LOD	4.68 ± 0.00 ^Ad^	3.48 ± 0.02 ^Bd^
	ALA5	<LOD	5.94 ± 0.01 ^Ac^	5.19 ± 0.03 ^Bc^
	GL10	<LOD	7.05 ± 0.01 ^Ab^	6.83 ± 0.00 ^Bb^
	GLA5	<LOD	9.69 ± 0.04 ^Aa^	8.95 ± 0.03 ^Ba^
*Flavanols*				
Procyanidin A2	AL10	8.21 ± 0.02 ^Ab^	1.44 ± 0.01 ^Bc^	1.07 ± 0.01 ^Cc^
	ALA5	7.09 ± 0.01 ^Ad^	1.92 ± 0.01 ^Ba^	1.31 ± 0.05 ^Ca^
	GL10	7.44 ± 0.00 ^Ac^	1.21 ± 0.01 ^Bd^	1.03 ± 0.03 ^Cc^
	GLA5	9.16 ± 0.01 ^Aa^	1.57 ± 0.02 ^Bb^	1.18 ± 0.01 ^Cb^
Procyanidin B1	AL10	1.46 ± 0.01 ^Ad^	<LOD	<LOD
	ALA5	2.25 ± 0.02 ^Ac^	<LOD	<LOD
	GL10	7.85 ± 0.04 ^Aa^	2.71 ± 0.01 ^Bc^	<LOD
	GLA5	7.41 ± 0.03 ^Ab^	3.11 ± 0.04 ^Bb^	<LOD
Procyanidin B2	AL10	9.88 ± 0.01 ^Ad^	1.25 ± 0.03 ^Bd^	<LOD
	ALA5	13.23 ± 0.05 ^Ab^	2.33 ± 0.03 ^Bc^	1.47 ± 0.02 ^Cc^
	GL10	11.51 ± 0.01 ^Ac^	9.76 ± 0.08 ^Bb^	9.43 ± 0.10 ^Ca^
	GLA5	15.69 ± 0.02 ^Aa^	11.58 ± 0.05 ^Ba^	6.59 ± 0.05 ^Cb^
Catechin	AL10	<LOD	<LOD	<LOD
	ALA5	<LOD	6.61 ± 0.03 ^Aa^	1.03 ± 0.02 ^Ba^
	GL10	<LOD	<LOD	<LOD
	GLA5	<LOD	<LOD	<LOD
Epigallocatechin gallate	AL10	2.32 ± 0.02 ^Cc^	6.58 ± 0.01 ^Ba^	7.20 ± 0.03 ^Aa^
	ALA5	5.38 ± 0.05 ^Ba^	5.43 ± 0.06 ^Bb^	5.83 ± 0.03 ^Ab^
	GL10	1.69 ± 0.01 ^Cd^	4.44 ± 0.03 ^Bd^	4.84 ± 0.01 ^Ac^
	GLA5	3.37 ± 0.01 ^Bb^	4.71 ± 0.06 ^Ac^	4.78 ± 0.05 ^Ad^
Epicatechin	AL10	<LOD	3.21 ± 0.01 ^Aa^	<LOD
	ALA5	1.25 ± 0.02 ^Aa^	<LOD	<LOD
	GL10	<LOD	<LOD	<LOD
	GLA5	<LOD	<LOD	<LOD
*Flavonols*				
Quercetin 3-Glucoside	AL10	<LOD	<LOD	<LOD
	ALA5	1.28 ± 0.03 ^Aa^	1.20 ± 0.01 ^Ba^	<LOD
	GL10	<LOD	<LOD	<LOD
	GLA5	<LOD	<LOD	<LOD
Isorhamnetin	AL10	5.64 ± 0.05 ^Aa^	<LOD	<LOD
	ALA5	5.71 ± 0.03 ^Aa^	<LOD	<LOD
	GL10	<LOD	<LOD	<LOD
	GLA5	<LOD	<LOD	<LOD
*Antioxidant capacity*				
DPPH^•^ (μmol/g) ^1^	AL10	14.19 ± 0.52 ^Ab^	13.51 ± 0.20 ^Bb^	14.10 ± 0.52 ^Ab^
	ALA5	8.29 ± 0.43 ^Bd^	5.70 ± 0.16 ^Cd^	9.87 ± 0.41 ^Ad^
	GL10	10.59 ± 0.25 ^Ac^	9.14 ± 0.32 ^Bc^	10.77 ± 0.11 ^Ac^
	GLA5	15.47 ± 0.26 ^Aa^	14.50 ± 0.21 ^Ba^	15.51 ± 0.28 ^Aa^
ABTS^•+^ (μmol/g) ^1^	AL10	16.89 ± 0.18 ^Bd^	19.97 ± 0.46 ^Ac^	22.08 ± 0.35 ^Ab^
	ALA5	20.96 ± 0.49 ^Bb^	18.24 ± 0.37 ^Cd^	28.79 ± 0.22 ^Aa^
	GL10	18.50 ± 0.51 ^Cc^	21.05 ± 0.26 ^Ab^	19.43 ± 0.28 ^Bc^
	GLA5	23.94 ± 0.22 ^Ca^	24.69 ± 0.33 ^Ba^	28.50 ± 0.15 ^Aa^
FRAP (µmol FeSO_4_/g)	AL10	13.60 ± 0.36 ^Ad^	11.60 ± 0.19 ^Bc^	13.40 ± 0.20 ^Ad^
	ALA5	19.70 ± 0.44 ^Cb^	28.40 ± 0.27 ^Aa^	24.30 ± 0.61 ^Ba^
	GL10	18.50 ± 0.15 ^Bc^	20.70 ± 0.18 ^Ab^	15.30 ± 0.52 ^Cc^
	GLA5	27.70 ± 0.23 ^Ba^	28.40 ± 0.15 ^Aa^	19.80 ± 0.45 ^Cb^

AL10: acerola + *L. paracasei* L10; ALA5: acerola + *L. acidophilus* LA-05; GL10: guava + *L. paracasei* L10; GLA5: guava + *L. acidophilus* LA-05. A–C: different superscript capital letters in the same row denote differences (*p* ≤ 0.05) based on the Student’s *t*-test or Tukey’s test; a–d: different superscript small letters in the same column for the same time interval and measured parameter denote differences (*p* ≤ 0.05) based on the Student’s *t*-test or Tukey’s test. ^1^ The results are expressed as micromoles of Trolox equivalent antioxidant capacity per gram of sample (µmol/g). Abbreviations: ABTS**^•+^**, cation—2,2-azino-bis (3-etilbenzo-tiazoline)-6-sulfonic acid; DPPH**^•^**, cation—2,2-diphenyl-1-picrylhydrazyl; FRAP, ferric reducing antioxidant power; FeSO_4_, ferrous sulfate < LOD: below the limit of detection.

## Data Availability

All data generated or analyzed during this study are included in this published article.

## Supplementary Materials

The following supporting information can be downloaded at https://www.mdpi.com/article/10.3390/foods13091375/s1, Table S1: Physicochemical parameters of freeze-dried fruit processing co-products used in assays to evaluate potential prebiotic properties (Araújo et al., 2020), and Table S2: Identification of metabolites by ^1^H-NMR in media with fermented acerola and guava fruit co-products (AL10, ALA5, GL10, and GLA5), fructooligosaccharides (FOS), and negative control (NC; without fermentable substrate) at time zero (baseline) and 48 h of in vitro fecal fermentation.
