# Supplementary material for: Fermenting Acerola (Malpighia emarginata D.C.) and Guava (Psidium guayaba L.) Fruit Processing Co-Products with Probiotic Lactobacilli to Produce Novel Potentially Synbiotic Circular Ingredients
Source: Foods. 2024 Apr 29;13(9):1375. doi: 10.3390/foods13091375 (PMC11083529; doi:10.3390/foods13091375)
Supplement: Supplementary file 1 [file foods-13-01375-s001.zip › foods-2974191-supplementary.pdf]

Table S1. Physicochemical parameters of freeze-dried fruit processing co-products used in assays to evaluate potential prebiotic properties (Araújo et al., 2020).

**Parameters**	**Fruit processing by-products**	
	Acerola	Guava
Fructose (g/100 g)	8.48 ± 0.01^{A}	3.92 ± 0.01^{B}
Glucose (g/100 g)	5.31 ± 0.01^{A}	3.17 ± 0.01^{B}
Maltose (g/100 g)	1.52 ± 0.01^{A}	1.53 ± 0.01^{A}
Soluble fiber (g/100 g)	8.09 ± 0.69^{B}	33.44 ± 3.63
Insoluble fiber (g/100 g)	61.16 ± 1.75^{A}	49.12 ± 1.58^{B}
Total dietary fiber (g/100 g)	69.25 ± 1.06^{B}	82.55 ± 2.05^{A}
Total flavonoids (mg EC/100 g)[1]	79.83 ± 0.23^{A}	44.09 ± 1.01^{B}
Total phenolics (mg EAG/100 g)[2]	492.107 ± 0.54^{A}	304.057 ± 0.94^{B}

A-B: Different superscript capital letters in the same row denote difference ($p \leq 0.05$), based on Tukey's test. among co-products, based on Student's t-test. [1]The results are expressed in milligram equivalents of catechin (EC) per hundred grams of sample (mg EC/100 g). [2]The results are expressed in milligram equivalents of gallic acid (EAG) per hundred grams of sample (mg EAG/100 g).

Table S2. Identification of metabolites by ^{1}H-NMR in media with fermented acerola and guava fruit co-products (AL10, ALA5, GL10, and GLA5) and fructooligosaccharides (FOS), as well as in negative control (NC; without fermentable substrate) at time zero (baseline), and 48 h of *in vitro* fecal fermentation.

Number	Chemical constituents	NC		FOS		ALA5		AL10		GLA5		GL10	
		0 h	48 h	0 h	48 h	0 h	48 h	0 h	48 h	0 h	48 h	0 h	48 h
1	Biliary salts	x	x	x	x	x	x	x	x	x	x	x	x
2	2-methylbutyrate	x	x	x	x	x	x	x	x	x	x	x	x
3	Valerate	x	x	x	x	x		x	x	x	x	x	x
4	N-butyrate	x	x	x	x	x		x	x	x	x	x	x
5	Leucine	x		x	x	x		x		x		x	
6	Isoleucine	x		x	x	x		x		x		x	
7	Valine	x		x	x	x		x		x		x	
8	Propionate/ propionic acid	x		x	x	x		x		x		x	
9	Isobutyrate/ butyric acid	x		x	x	x		x		x		x	
10	3-methyl-2-oxoisovalerate	x		x	x	x	x	x	x	x	x	x	
11	2-oxoisovalerate	x		x	x	x	x	x	x	x	x	x	
12	Ethanol	x	x	x	x	x	x	x	x	x	x	x	x
13	3-hydroxybutyrate	x	x	x	x	x	x	x	x	x	x	x	x
14	Threonine						x		x		x		x
15	Lactate/lactic acid	x	x	x	x	x	x	x		x		x	
16	Alanine	x	x	x	x	x	x	x	x	x	x	x	x
17	Lysine	x	x	x	x	x	x	x	x	x	x	x	x
18	Ornithine	x	x	x	x	x	x	x	x	x	x	x	x
19	Acetate/acetic acid	x	x	x	x	x	x	x	x	x	x	x	x
20	Proline	x	x	x		x	x	x	x	x	x	x	x
21	Glutamate	x	x	x		x	x	x	x	x	x	x	x
22	5-aminopentanoate	x		x		x	x	x	x	x	x	x	x
23	Succinate/succinic acid	x			x	x	x	x	x	x	x	x	x
24	Methylamine	x	x	x	x	x	x	x	x	x	x	x	x
25	Methionine	x		x		x	x	x		x		x	x
26	Citrate/citric acid	x		x		x	x	x		x		x	x
27	Aspartate	x		x				x		x		x	x
28	Asparagine	x	x	x	x	x		x		x		x	x
29	Trimethylamine	x	x	x	x	x	x	x	x	x	x	x	
30	Putrescine	x	x	x	x	x	x	x	x	x	x	x	x

31	Malonate	x	x	x	x	x	x	x	x	x	x	x	x
32	Glycine	x	x		x								
33	Fructose	x	x	x	x	x	x	x	x	x	x	x	x
34	Dihydroxyacetone	x	x	x	x	x	x						
35	α-Xylose	x				x	x	x	x	x	x	x	x
36	β-Xylose	x				x	x	x		x		x	
37	β-Glucose	x				x	x	x		x		x	
38	α-Glucose	x				x		x		x		x	
39	β-galactose	x				x	x	x		x		x	
40	UDP-glucuronate	x	x	x		x		x		x	x	x	x
41	Homovanillate	x		x	x	x	x	x	x	x	x	x	x
42	3-hydroxyphenylacetate							x		x		x	
43	P-cresol	x	x	x		x	x	x	x	x	x	x	x
44	Tyrosine	x	x	x		x	x	x	x	x	x	x	x
45	5-aminosalicylate	x		x		x	x	x	x	x	x	x	x
46	Phenylalanine	xx	x	x		x	x	x	x	x	x	x	x
47	Uracil	x	x	x		x	x	x	x	x	x	x	x
48	N-acetyl-5-aminosalicylate	x	x	x		x	x	x		x		x	
49	Phenylacetate	x	x	x		x	x	x		x		x	
50	Tryptophan	x						x		x		x	
51	Hypoxanthine	x		x		x		x		x		x	
52	Formate/formic acid	x	x	x		x	x	x	x	x	x	x	x
53	Caprylate	x		x	x	x	x	x	x	x	x	x	x
54	Isocaproate	x	x	x		x	x	x	x	x	x	x	x
55	Isovalerate	x		x	x	x	x	x	x	x	x	x	x
56	3-hydroxyisovalerate						x		x		x		x
57	Total lipids	x	x	x	x	x	x	x	x	x	x	x	x
58	Gamma-aminobutyric acid (GABA)	x	x	x	x	x	x	x	x	x	x	x	x
59	Ketoisovalerate	x		x	x	x	x	x	x	x	x		x
60	Acetone	x		x		x	x	x	x	x	x	x	x
